# Cotranscriptional Chromatin Remodeling by Small RNA Species: An HTLV-1 Perspective

**DOI:** 10.1155/2012/984754

**Published:** 2012-02-09

**Authors:** Nishat Aliya, Saifur Rahman, Zafar K. Khan, Pooja Jain

**Affiliations:** Department of Microbiology and Immunology, Drexel Institute for Biotechnology and Virology Research, Drexel University College of Medicine, 3805 Old Easton Road, Doylestown, PA 18902, USA

## Abstract

Cell type specificity of human T cell leukemia virus 1 has been proposed as a possible reason for differential viral outcome in primary target cells versus secondary. Through chromatin remodeling, the HTLV-1 transactivator protein Tax interacts with cellular factors at the chromosomally integrated viral promoter to activate downstream genes and control viral transcription. RNA interference is the host innate defense mechanism mediated by short RNA species (siRNA or miRNA) that regulate gene expression. There exists a close collaborative functioning of cellular transcription factors with miRNA in order to regulate the expression of a number of eukaryotic genes including those involved in suppression of cell growth, induction of apoptosis, as well as repressing viral replication and propagation. In addition, it has been suggested that retroviral latency is influenced by chromatin alterations brought about by miRNA. Since Tax requires the assembly of transcriptional cofactors to carry out viral gene expression, there might be a close association between miRNA influencing chromatin alterations and Tax-mediated LTR activation. Herein we explore the possible interplay between HTLV-1 infection and miRNA pathways resulting in chromatin reorganization as one of the mechanisms determining HTLV-1 cell specificity and viral fate in different cell types.

## 1. Introduction

In the myriad interactions between viruses and host cells, there is a constant struggle for survival that causes both sides to adopt strategies counteracting each other's effect. More often than not, the error-prone replication of viruses offers them an advantage of selective pressure enabling them to accumulate genetic mutations over time that helps evade host immune defense mechanisms. Most chronic viruses seem to have an edge in this struggle in that they evolve means to manipulate and exploit host molecular pathways to persist in the hostile cellular environment and remain hidden from immune surveillance [1]. In this regard, retroviruses have succeeded in establishing latent infection and developing drug resistance through escape mutants like very few other chronic viruses. One of the strategies utilized by retroviruses is the modulation of chromatin structure and regulation of the rate at which transcription occurs in the target cell. Chromatin remodeling in the context of retroviral infection is being explored as a potent means of long-term persistence.

Many studies have shown that the exercise of chromatin modulation in retroviral infection begins with the proviral integration into the host genome [2]. The site at which this integration occurs is important as it determines the kind of chromatin remodeling that the virus might cause and the rate at which viral proteins are produced. This in turn determines if the viral infection becomes latent or remains active. Persistence, as demonstrated by latent viruses, is thus largely dictated by the nature of virally encoded integrase enzyme. It requires the provirus to integrate into a site that is transcriptionally inactive or less active so that there is minimal viral gene expression. Conversely, a productive infection is a result of integration into transcriptionally active regions on the host genome resulting in a higher rate of viral protein expression [1]. Human T cell leukemia virus 1 (HTLV-1), a deltaretrovirus, behaves preferentially in the former fashion by altering chromatin structure to remain latent and thus aid in its survival and persistence [3]. In addition, methylation along the 5′ long terminal repeat (LTR) region of the virus contributes to regulation of viral persistence [4].

HTLV-1, the first retrovirus to be associated with human malignancies, is the causative agent of adult T cell leukemia (ATL) and HTLV-1 associated myelopathy/tropical spastic paraparesis (HAM/TSP) [5]. The virus has a propensity for infecting CD4^+  ^T cells [6] with CD8^+  ^T cells serving as reservoirs [6]. Other secondary cell types such as CD8^+^ T cells [7], cells of the monocyte-macrophage lineage, and dendritic cells [8] as well as those belonging to the resident CNS cell population [9] are also known to be infected. One of the factors to be considered during this observation is that some of the cell types refractile to viral transcription also tend to express lower levels of miRNA processing proteins.

A number of independent studies have identified integration sites of HTLV-1 in the human genome [10–13]. Derse et al., in 2007, mapped 541 integration sites of the virus in HeLa cells comparing them to other retroviral integration sites and showed that integration does not correspond merely to transcriptional units and transcriptional start sites. Rather, the apparent nonrandom site integration is monoclonal in nature [14] and predominantly reliant on the structure and/or sequence of viral integrase enzyme [13]. A clear demarcation appears to exist between the integration preferences of HTLV-1 in carrier cells versus leukemic cells. HTLV-1 integrates into nontranscribing heterochromatin alphoid repeats in carrier cells, while in leukemic cells, it preferentially integrates at actively transcribing DNA units [10].

Once integration has occurred, viral replication and successful infection among other factors depend on Tax, the virally encoded transactivator protein largely involved in cellular transformation. A major regulatory function of the retroviral transactivating protein is its ability to interfere with the host cellular miRNA machinery [15, 16]. Altered miRNA expression profiles have been observed between retrovirus-infected and uninfected cells that can also be associated with disease progression and development of cancer [17]. In the context of HTLV-1 infection, a number of recent studies have identified distinct miRNA patterns in infected cells that progress to ATL [18–20]. Although results of the individual studies have disparities between them, all of them identified the tumor suppressor gene TP531NP1 to be commonly repressed in infected cells [21]. A more detailed analysis of miRNA and its differential expression in HTLV-1-infected cells will be discussed in a later section of the paper.

Besides the canonical role of miRNAs as translational repressors of mRNA expression, emerging evidence indicates a significant modulatory role for miRNA at the level of chromatin [22, 23]. The miRNAs accomplish this either through direct methylation along the promoter region of specific genes or more indirectly through the epigenetic modification of histone proteins surrounding the chromatin of the target region [24]. This phenomenon referred to as RNA-induced initiation of transcriptional silencing (RITS) [25, 26] has been implicated in the regulation of a number of human genes [27]. Kim et al. demonstrated that endogenous miRNA recruit argonaute 1 (Ago1), EZH2, a PcG member, and H3K27me3 to the promoter region of the target gene and suppress its expression [28]. In case of HIV-1, another human retrovirus, the proviral promoter is also influenced by RITS in infected cells [29]. Retrovirus-derived miRNAs like those generated by processing of the TAR element of HIV-1 appear to be involved in RNAi-mediated Transcriptional Gene Silencing (RNAi-TGS). Several small ssRNA species cleaved from the TAR region by Dicer have been implicated in dampening cellular as well as viral gene transcription and promoting viral latency [30–32]. Taken together, it is possible that RNAi-TGS keeps viral transcription in check through the recruitment of RITS at the viral promoter [33].

A similar mechanism of gene silencing induced by siRNA is quite likely in HTLV-1-infected cells. From previous studies, it is clear that expression levels of Tax responsive miRNA in infected cells is to a large extent modified through the NF*κ*B pathway [34]. Given that Tax is associated with a number of other cellular transcription factors like CREB, Ap-1, Myc, NFAT, SRF, p53, TGF-*β*, and so forth, that also regulate siRNA expression levels, its involvement in regulating RITS through these pathways cannot be ruled out. Since Tax is predominantly nuclear, one possible mechanism by which it might influence siRNA-mediated chromatin remodeling could be through sequestering mature siRNA from its target sequence on the chromatin, preventing its regulation. Alternatively, Tax could also interact with the ribonuclease III enzyme Drosha within the nucleus, modulating its function and preventing it from cleaving out the precursor miRNA element from the primary miRNA transcript. Besides the nucleus, Tax occupies a number of subcellular sites in the infected cell. Although mechanistically poorly understood, it is known to shuttle to the cytoplasm from the nucleus, a process that was demonstrated through a heterokaryon fusion assay [35]. Also, there have been studies questioning the nuclear assembly of Tax with its transcriptional activating counterparts—CBP/p300 and RelA, proposing a possible cytoplasmic assembly [35, 36].

**Figure 1:**
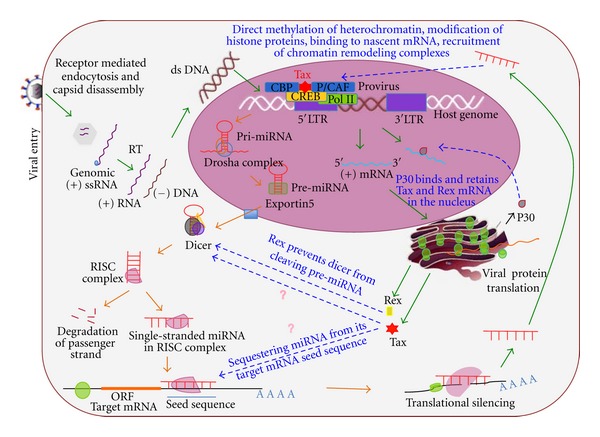
The interaction between HTLV-1 infection pathway and miRNA pathways. HTLV-1 infection begins with viral gp46 and gp21 envelop proteins recognizing and binding receptors on target cell membrane followed by envelop fusion and receptor-mediated endocytosis. Once inside the cell, HTLV-1 loses its capsid and releases the single-stranded (+) ve sense RNA into the cytoplasm that undergoes reverse transcription to (−) ve sense DNA. A second DNA strand is then formed; the two strands form a helix, enter the nucleus, and integrate with the host genome forming the proviral particle. Transcription ensues with cellular pol II, CREB, CBP, p300, P/CAF, and viral Tax assembling at the 5^′  ^LTR of the promoter region. This leads to Tax-dependent transcription of viral mRNA, transport of mRNA into the cytoplasm and its subsequent translation on ribosomes. The continuous green arrows indicate the HTLV-1 infection pathway, while the orange arrows indicate the miRNA biogenesis and mechanism of action pathway. The discontinuous blue arrows indicate points at which viral proteins might interact with the miRNA pathway and also where miRNA might interfere with viral transcription. Chromatin remodeling is one of the interfering mechanisms that can be brought about by siRNA direct binding of siRNA coupled with the RISC complex to DNA, methylation of heterochromatin regions of the chromosome, or modification of histone proteins associated with chromatin.

In addition, HTLV-1 Rex protein has been demonstrated to interact with Dicer and block it from processing mature miRNA from precursor miRNA and suppressing its RNA silencing activity [37]. The subsequent sections of the paper focus on HTLV-1 infection and the role of Tax protein in disease progression. Further, various small RNA pathways and their capacity to influence chromatin remodeling are described. Finally, a more detailed description is presented on interaction of viruses with miRNA in general and retrovirus and HTLV-1 in specific proposing probable points of intersection that can result in miRNA-mediated chromatin reorganization as depicted in Figure 1.

## 2. HTLV-1 and Its Transactivator Protein Tax

The virus predominantly gets transmitted through cell-cell contact, prompting the formation of a microtubule-organizing center (MTOC) oriented towards the virological synapse [38]. At the synapse, viral RNA and Gag protein accumulate at the MTOC and get transported into the uninfected cell [39]. Tax plays a role in synapse formation, MTOC orientation, and intracellular adhesion molecule −1 (ICAM-1) engagement with lymphocyte function associated antigen −1 (LFA-1) [40]. Although it was previously believed that cell-free HTLV-1 is largely noninfectious, there is emerging evidence that HTLV-1 can enter naïve cells through receptor-mediated endocytosis. The human glucose transporter GLUT-1 [41] and surface heparin proteoglycan [42] have been identified as possible receptors for cell-free virus.

HTLV-1 is a relatively complex retrovirus on account of the fact that in addition to the retroviral structural *gag, pol,* and *env *genes flanked by 3′ and 5′ LTR regions, it has a *pX* region located between the 3^′  ^LTR and *env* genes encoding Tax, Rex, and other accessory proteins [43]. Tax is encoded by open reading frame (ORF) IV of the *pX *region and is principally functional in the nucleus. Through DNA array studies, expression profiles of more than 300 of the ~2000 cellular genes assayed were found to be significantly altered under the influence of Tax [44] acting through several pathways as previously mentioned. As an oncoprotein, the most critical function of Tax appears to be cell survival, proliferation, and ultimately transformation of the cell into an ATL state. Oncogenicity is most often associated with genotypic and phenotypic instability of transformed cells. Genomic instability in HTLV-1-induced leukemia is thought to be caused by Tax in two phases:firstly, by inhibition of cellular DNA repair pathways and secondly by the loss of cell cycle checkpoint controls [43, 45, 46].

The 40-kDa Tax protein is essentially involved in HTLV-1 gene expression from three 21-bp Tax responsive elements (TRE) located within the U3 region of the viral promoter [47, 48]. Each TRE is composed of domains A, B, and C, but the central B region is a conserved 8-nucleotide (nt) core sequence (TGACGTCA) that closely mimics a cyclic AMP (cAMP) responsive element (CRE) and is flanked by 5′ and 3′ G/C-rich sequences [49]. Tax activates transcription by recruiting the cellular transcription factors—CRE binding protein (CREB) and serum response factor (SRF or p67^SRF^) to the CRE [50, 51]. Tax interacts with dimeric CREB [52] as a homodimer forming a ternary complex that in turn helps to stabilize the CREB/TRE complex [53]. Once stabilized, Tax then independently recruits the two cellular coactivators-p300/CREB-binding protein (p300/CBP) and p300/CBP-associated factor (P/CAF), both of which bind to two distinct regions in the amino-terminus and carboxyl-terminus of Tax, respectively, and eventually activates transcription by histone acetylation through chromatin remodeling [54–56]. In addition, Tax has shown to reduce histone protein and transcript levels in HTLV-1 infected compared to uninfected T cell lines [57]. The protein also influences transcription of a number of cellular promoters, namely, IL-2, IL-13, IL-15, IL-2R, c-Fos, GM-CSF, and so forth [58–63]. Modulation of cellular gene expression is through several cellular signaling cascades—four of these cardinal pathways include CREB-ATF [64], NF*κ*B [65], AP-1 [66], and SRF [67]. Regulation of these pathways by Tax has been extensively reviewed elsewhere [68]. One major outcome is the quelling of the tendency of virus-infected cells to undergo apoptosis and senescence [69, 70]. In addition, Tax represses DNA damage control checkpoints and also activates several proliferative factors that facilitate progression of cell cycle into the replicative phase, enhancing cell division [71].

Members of the stimulatory protein (Sp1 and Sp3) family of transcription factors physically interact with the GC regions within TRE-1 repeat III, and purified Sp1 protein competes with purified CREB protein for binding to this site, but in the presence of Tax purified Sp1 can form a protein complex with Tax and CREB [72]. In cells of the monocytic-macrophage lineage (secondary target cell population), factors belonging to the activator protein (AP-1) family of basic region/leucine zipper (bZIP) proteins (Fra-1, Fra-2, JunB, and JunD) are shown to be upregulated [73, 74] and bind to the TRE-1 repeat II site thereby activating basal-and Tax-mediated transactivation of the LTR [75]. However, in the same cell type lineage, another family of bZIP factors, CCAAT/enhancer binding protein (C/EBP), promotes low level of viral gene expression in the absence of Tax (C/EBP*β*, C/EBP*δ*, C/EBP*ε*), while in the presence of Tax it (C/EBP*α* and C/EBP*β*) inhibits high level of viral gene expression [76]. Other transcription factors like members of the CREB family (CREB-2-activating transcription factors—ATF-1 and ATF-2) [77, 78] and the histone deacetylase HDAC1 [77] have also been identified in the LTR complex [79]. The TORC family of transcriptional regulators (viz., TORC1, TORC2, and TORC3) are coactivators of Tax protein and the removal of these factors inhibits Tax activity. The cofactor p300 further enhances for TORC activity [80, 81].

However, majority of these investigations highlighting the importance of the cellular transcription factors (CREB, Sp1, Sp3, AP-1, C/EBP, p300/CBP, and P/CAF) in HTLV-1 Tax-mediated LTR activation [50, 82–85] and the ability of Tax protein to interact with these factors independently [54, 86, 87] have been carried out using transiently transfected viral reporter plasmids or in cell lines that otherwise are not the primary target for HTLV-1* in vivo*. Studies with HIV-1 have shown that the integrated provirus differs from a transfected viral plasmid both physically [78] and also in the requirement of certain cellular factors especially those belonging to the chromatin-remodeling histone acetyltransferase (HAT) family [88–90] or even in the transcriptional repressor domain [91]. It demonstrates that transient transfection cell systems do not convey the real picture by undermining the crucial role of chromosomal structure in transcriptional regulation. In order to gain a better understanding of viral gene regulation as well as the complex interplay between the integrated provirus, host cellular transcription factors, the viral transcription transactivating protein, and the cellular siRNA machinery during the course of infection and reactivation following latency, it would certainly be more realistic and physiologically relevant if such studies are carried out with stably integrated viral LTR in a clinically relevant cell type that is formatted in the context of cellular chromatin. To this end, we generated HTLV-1 LTR stable integrants with a reporter luciferase gene (HTLV-1 LTR-luc) in the Jurkat cell line, representative of the natural target CD4^+^ T-cell population, to characterize realistically the intricacies involved in the interplay between the integrated provirus, cellular transcription factors, and the viral transactivating protein Tax (Rahman et al., unpublished data). To investigate the comparative activation/repression of cellular transcription factors between stably integrated and transiently transfected HTLV-1 LTR in at least one native target cell phenotype, both in the absence and presence of Tax, a high-throughput analysis of such factors was performed using protein-DNA array technology. Many substrates and factors associated with the two major chromatin-remodeling complexes, SWI/SNF and HATs, were activated in the stably integrated clones following transfection with Tax. To explore the observed heightened activation of factors necessary for chromatin remodeling complexes, we explored the upstream miRNA regulatory pathway by the microarray approach. A global downregulation in the expression of cellular miRNAs in the HTLV-1 LTR-luc stably integrated CD4^+^ T-cell clone was observed in the presence of Tax, implying the ability of Tax to modulate the cellular miRNA machinery. When compared to results presented with the transcription factor array, many of the downregulated miRNAs were found to target the mRNA coding for the P/CAF and p300 HAT family members, suggesting a role for Tax in downregulating the expression of cellular miRNAs that are in turn involved in suppressing the expression of transcription factors involved in chromatin remodeling. The results demonstrate that Tax can modulate the cellular miRNA machinery and downregulate the expression of miRNAs identified to be involved in regulating the translation of chromatin-remodeling HAT factors (Rahman et al., unpublished data). Given the rising importance of siRNA-mediated modulation of gene expression in a viral infection context, it would be interesting to explore how HTLV-1 alters the siRNA/miRNA in its primary target cell. Small noncoding RNA species make up the bulk of cellular RNA, and their regulatory potential is increasingly being recognized as significant and proportional to their presence in the cell. Their regulatory potential encompasses chromatin reorganization and the following section of the review focuses on giving a brief description of various noncoding small RNA molecules and their possible involvement in transcriptional modulation through chromatin.

## 3. Small Interfering RNA and Gene Silencing

Small noncoding RNA species are rapidly being recognized as a significant influence on gene expression and functioning of cells. RNA silencing pathways have been traditionally classified based on the mechanism of action, intracellular location, and the class of RNA molecule involved. There are similarities in the organization of some of the components in these pathways, and an inevitable intersection exists between them in some instances [92]. Three major classes of RNA have been annotated to be involved in modulating cellular gene expression namely, small interfering RNA (siRNA), micro-RNA (miRNA), and piwi-associated RNA (piRNA). While siRNA and piRNA have an equally potential role in posttranscriptional as well as transcriptional gene repression, miRNA was until recently predominantly descried for its cytoplasmic role of mRNA suppression [93]. From current studies, a transcriptional chromatin-modulating role for miRNA is emerging and gaining importance especially in the context of a viral infection [23].

Small interfering RNAs are ~21 nt single-stranded RNA molecules cleaved out of larger dsRNA that can either be of endogenous or exogenous origin. siRNA identify their target through complete and perfect sequence complementarity and silence target mRNAs through complementary binding [94]. Some organisms have developed mechanisms to amplify their siRNA after target recognition through the expression of an RNA dependent RNA polymerase (RdRP). The RdRP enzyme either by itself produces new single stranded siRNA or amplifies ssRNA into dsRNA that is then cleaved by Dicer to generate mature siRNA [95].

miRNAs are ~19–24 nt short non-coding RNA that modulate ~60% of all human protein coding cellular genes, successfully modifying the outcome of various microbial infections and disease states [96]. Typical miRNA biogenesis is initiated with the RNA pol II mediated transcription of long primary miRNA (pri-miRNA) that contain one or more ~80 nt hairpin (stem-loop) structures. The pri-miRNA is processed by an RNase III enzyme Drosha, which, along with its coeffector DGCR8, recognizes and cleaves ~22 bp down the stem yielding a precursor miRNA (pre-miRNA) approximately 60 nt in length comprising 2-nt 3′ overhangs. The pre-miRNA is transported into the cytoplasm aided by Exportin 5 through the nuclear pore complex. A second RNase III enzyme Dicer in association with Tar RNA binding protein (TRBP) cleaves the terminal loop structure of the pre-miRNA, generating a ~22-bp duplex [97, 98]. One of the strands of the duplex associates with an RNA-induced silencing complex (RISC) functioning as a guide to the target mRNA “seed” sequence, while the other passenger strand gets degraded. The RISC complex is composed of the Argonaute family of proteins (Ago), some of which have endonuclease activity and enzymatically cleave the target mRNA. In addition, Ago proteins guide the complex to the target site and also aid in the degradation of the passenger strand [99]. The following section deals with the involvement of these small non-coding RNA in TGS through reorganization of nuclear chromatin.

## 4. Chromatin Remodeling

The term refers to the effective shifting of nucleosome core along the length of the DNA molecule [100]. This shift in many cases results in the physical disassembly and reassembly of the nucleosome core and requires the involvement of ATPase containing complexes. The four known ATPase complexes associated with chromatin remodeling are SWI2/SNF2 (mammalian Brm (SNF2*α*) and Brg1 (SNF2*β*)), ISWI (imitation switch), Mi-2 (CHD1), and INO80 [101]. During processes such as DNA replication and transcription, the state of chromatin organization and the positioning pattern of associated histone proteins are critical rate determining factors. A large number of histone-modifying enzymes and factors get associated with histones and alter their state to affect winding and unwinding of chromatin DNA as required by the specific cell.

### 4.1. Chromatin Remodeling by siRNA and miRNA

Heterochromatin reorganization by miRNA is a mechanism that has been generally described to be a result of association of siRNA/miRNA with the RNA-induced initiation of silencing [26] complex, which was first described by Verdel et al. in 2004 [25]. RITS is a multiprotein complex consisting of components that aid binding with RNA and chromatin simultaneously. It has a chromodomain protein, Chp1, that is known to interact with centromeres of chromosomes [102] and an argonaute family protein, Ago1, with endonuclease activity that can bind small RNA. In addition, a recently identified Tas3 protein is present in the RITS complex that is yet to be functionally characterized. Sequences homologous to the *dg* and *dh* region of centromeres were found on siRNA copurified with RITS complexes, indicating plausible complementary base pairing of RITS-associated siRNA with centromeric regions of the chromosome. Also, all three proteins of the complex are essential requirements for H3K9 methylation as well as centromeric chromatin-binding Swi6p protein known to cause heterochromatic gene silencing [102].

It has also been proposed that an alternate mode of interaction could be between RITS-associated siRNA and nascent immature mRNA transcribed from the H3K9 centromeric chromatin region. An interesting outcome associated with this phenomenon is that it serves as a self-amplifying mechanism for siRNA. The inhibition of mRNA from maturing and its subsequent degradation could be the source of new siRNA with a sequence complementary to that of the H3K9 region of the centromere, thus augmenting gene silencing [103]. Although siRNAs with sequences homologous to LTR regions have not been clearly identified, RITS-complex-associated silencing of LTR-associated genes cannot be ruled out. One reason being that LTR-associated gene silencing requires the presence of Ago1 [104] that is generally found in affiliation with other small RNA processing protein complexes namely, RISC and RITS. Also, the relative abundance of siRNA directed against LTR regions might be lower and less obviously discernable compared to *dg* and *dh* regions of centromeres. Since virally encoded proteins directly and indirectly get associated with various stages of the RNAi biogenesis and functioning pathways, it is reasonable to speculate that viral proteins might influence heterochromatin modeling and expression states.

## 5. Viral miRNAs and Virus-Induced Modulation of Cellular miRNA Pathway

### 5.1. miRNA of Viral Origin

A number of interactive stages and mechanisms have been described and proposed to elucidate the interplay between viruses and siRNA pathways. Besides cellular miRNAs, viruses also encode their own species of miRNA that modulate cellular processes favoring efficient viral proliferation and persistence. Herpesviruses were among the first to be shown to express miRNAs [105] which play significant roles in pathogenesis of the virus. For instance, HSV2 expresses miR1 that is a key modulator of ICP34.5 expression and hence progression of neuroinflammatory disease [106].

### 5.2. Retroviral miRNA

The idea of retroviruses coding for miRNA is in a way counterintuitive in that a cytoplasmic replication will likely yield miRNA precursors that would not be available for processing by the nuclear Drosha complex [107]. Nonetheless, many recent studies revealed retroviral-derived miRNA in the context of HIV-1 infection. Omoto et al. identified several miRNA originating from the nef region that affected transcription from the viral HIV-1 LTR promoter [108]. More recently, it was found that the HIV-1 TAR element is processed by Dicer to yield viral miRNA that could be detected in infected cells and apparently contribute to latency [30]. HTLV-1-encoded miRNA has been relatively less explored, and there is currently no evidence of the virus expressing any miRNA. Li et al., in 2008, performed an extensive computational sequence analysis to identify stem-loop structures resembling those harbored by pri-miRNA and pre-miRNA that could possibly be cleaved to generate microRNAs [109]. Ten such sequences were identified and when aligned with the HTLV-1 genome, it was predicted that two miRNAs could be produced from the plus strand transcript and 4 from the minus strand [110]. Upon further bioinformatics-aided searches of all the sequence reads against the HTLV-1 genome, both sequences mapped to transcribed regions on the plus strand [110]. More extensive studies will be required to investigate if the HTLV-1 genome can act as a source of miRNA.

### 5.3. Modulation of miRNA Pathway by Viruses

Viruses have been known to exploit cellular miRNAs to induce cell proliferation, suppress apoptosis, influence metabolic pathways, and finally bring about cellular transformation [111]. Increased expression of several oncogenic miRNA in response to retroviral infection has been previously documented [112–114]. There are several instances where viral proteins actively interact with RNA and alter their functioning in infected cells. The nonstructural (NS-1) protein of human influenza A virus has an RNA-binding domain that can associate with a number of RNA species causing RNA silencing suppressor activity [115, 116]. NS1 localizes both in the cytoplasm and the nucleus where it sequesters immature and mature forms of small interfering RNA, this activity is more evident in plants and needs to be elucidated further in mammals [117]. The HCV core protein predominantly localizes in the cytoplasm and interacts with Dicer through its N-terminal, lowering its miRNA processing efficiency. In addition, the HCV envelop protein E2 interacts with argonaute 2 (Ago2) inactivating it. Ago2 is part of the protein complex essential for generation of mature and active miRNA particles [118]. The Ebola virus non-structural protein VP35 consists of a dsRNA-binding domain similar to the NS1 of influenza A. It is generally found in cytoplasmic inclusion bodies and is proposed to sequester siRNA/miRNA preventing them from getting loaded on to RISC complexes, thus preventing them from directing mRNA silencing [117]. Adenoviruses use their dsDNA genome to encode two non-coding RNA molecules apart from viral proteins transcribed by RNA polymerase III. These VA1 RNA transcripts are produced at high levels in the nucleus of infected cells, saturating the Exportin 5 nuclear transport machinery. This in turn prevents precursor miRNA from being exported to the cytoplasm and mature into functional miRNA [119].

### 5.4. Modulation of miRNA Pathway by Retroviruses

**Figure 2:**
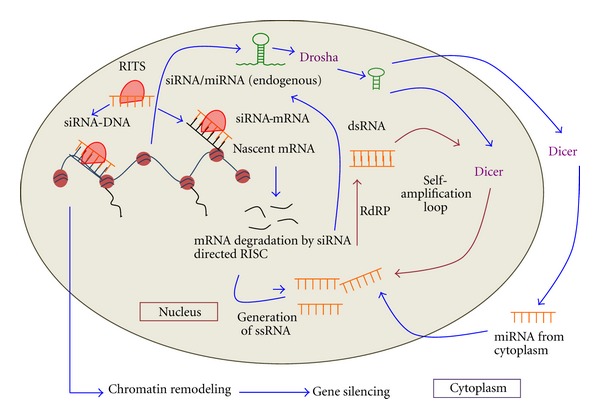
Mechanisms of siRNA-induced transcriptional silencing. miRNA/siRNA is imported into the nucleus from the cytoplasm after processing by Dicer through the nuclear pore complex. Alternatively, Dicer can also localize in the nucleus, allowing siRNA generation and amplification within the nucleus itself. Change in gene expression at the chromatin level directed by siRNA through the RNA-induced initiation of transcriptional silencing complex can be the outcome of a number of interactive mechanisms. Firstly, protein components of the RITS complex possessing both chromatin and RNA binding properties enable siRNA/miRNA to directly bind DNA. siRNA have homologous regions to the dg and dh regions of centromeric heterochromatin indicating a direct interaction between the two components. Secondly, miRNA can methylate promoter regions of genes or epigenetically cause the assembly of histone modifying proteins, changing the transcriptional state of chromatin. Thirdly, an interaction between siRNA and nascent mRNA transcribed from the centromeric region not only causes transcriptional repression of that region but also serves as an siRNA self-amplifying mechanism that uses degraded mRNA as a source of single-stranded RNA. Some of this ssRNA is amplified by RNA-directed RNA polymerase (RdRP) components which generate dsRNA further processed by nuclear Dicer into mature siRNA that augments silencing.

Retroviruses also modulate the host miRNA machinery in a number of ways. Primate Foamy Virus (PFV) primarily encodes a nuclear transactivator of transcription protein—Tas that through a yet unknown mechanism suppresses the effector function of miRNA [120]. HIV-1 Tat protein functions as a transactivator of transcription and is essential for viral replication and is also indicated in suppressing the RNAi pathway. While HTLV-1 Tax protein, which is also a transactivator [47] has not yet been shown to exhibit the similar function. Tat directly interacts with the helicase domain of Dicer and inactivates it, preventing cytoplasmic processing of miRNA [16, 48]. However, it is still not clear how the predominantly nuclear protein Tat interacts with the mostly cytoplasmic Dicer. One explanation could be that small levels of Tat could still remain cytoplasmic and interact with the RNAi pathway. Also, recently small levels of Dicer have been shown to be present in the nucleus where it also participates in processing miRNA and siRNA in association with RNA-dependent RNA polymerase (RdRP) (Figure 2). The Dicer processed TAR-derived miRNA of HIV-1 has been known to suppress RNAi through sequestration of TREB—a Dicer cofactor [121]. A similar possibility can be attributed to the Tax protein of HTLV-1, which is also mostly nuclear but could still interfere with the cytoplasmic phase of miRNA processing.

## 6. HTLV-1 Infection: Chromatin Remodeling via miRNA

### 6.1. miRNA Expression in Infected Cells

There have been independent studies linking the differential expression of miRNA profiles in HTLV-1-infected T cells with the progression to ATL. Pichler et al. (2008) performed RT PCR analysis on HTLV-1 transformed cell lines to determine differential expression in CD4^+^ T cells and Treg cells [19]. Seven miRNAs that had been previously shown to be associated with oncogenic transformation [20], miR-21, miR-24, miR-146a, miR-155, miR-191, miR-214, and miR-223 were considered, for this study as they are specific to Treg cells. Results demonstrated that miR-21, miR-24, miR-146a, and miR-155 were significantly upregulated in the HTLV-1-transformed cell lines, while miR-223 was downregulated. The miRNA profiling of PBMCs from acute ATL samples and HTLV-1 transformed cell lines revealed 6 miRNAs that were upregulated in both the ATL samples and the transformed cell line (i.e., miR-18a, 9, 17-3p, 130b, 20b, and 93), while 9 miRNAs were downregulated (i.e., miR-1, 130a, 199a, 126, 144, 335, 337, 338, and 432) [18]. Further miRNA-profiling studies were performed on ATL cells versus control PBMCs and CD4^+  ^T cells. Several miRNAs, namely, miR-150, miR-155, miR-223, miR-142-3p, and miR142-5p were upregulated and miR-181a, miR-132, miR-125a, and miR-146b were downregulated [20]. A similar miRNA profile was also seen in HTLV-1-infected non-ATL cells. miRNA 27-a controls the F-box protein FBW7/hCDC4-dependent cyclin E degradation by modulating ubiquitylation and turnover of cyclin E during specific stages of the cell cycle progression [122]. Not surprisingly, results from the above studies taken together indicate a highly variant miRNA profile owing to the cell type specificity of the virus and probably also to the differential expression of miRNA-associated cellular proteins.

### 6.2. Cellular Pathways

Cell longevity in HTLV-1 infection is mediated by Tax through two major cell-signaling pathways. In the first, the protein directly binds PI3K and promotes phosphorylation of Akt, which is a serine/threonine kinase that is involved in maintaining cell proliferation and survival through a series of downstream signaling events involving activating protein −1 (AP-1) [123] that is upregulated in ATL cells [124] as well as a large number of other cancer types. The second pathway involves binding of Tax to Ikk*γ* in the cytoplasm that triggers phosphorylation of Ikk*α* and Ikk*β* and a complex formation between the 3 Ikk components. The Ikk*α*-Ikk*β*-kk*γ* complex in turn phosphorylates I*κ*B*α* and releases it from NF*κ*B. Free NF*κ*B then migrates to the nucleus, activating the transcription of genes associated with the NF*κ*B responsive elements. A number of NF*κ*B target genes are known to enhance cell growth and proliferation and implicated in cancer development.

In spite of the fact that Tax is required for cell transformation and development of ATL, it is interesting to note that more than 50% of ATLs do not show Tax transcripts. In fact cells tend to evolve and accumulate mutations in Tax that silence its activity [39]. It is believed that the presence of Tax is needed for the emergence of ATL, but not for its maintenance. Since the oncoprotein is a major target of T lymphocytes, silencing it is a mechanism to subvert immune detection and clearance. It is quite possible that the maintenance of an active leukemic cell state is mediated through miRNA that are known to be significantly differentially regulated in ATL cells. Expression profile studies suggest that many of these miRNAs are associated with Tax levels in early ATL developmental stages. Chromatin reorganization as previously mentioned is influenced by miRNA and hence the rate of active viral transcription and maintenance of productive/latent viral state could be affected by the miRNA profile of the infected cell. Several of these miRNAs are associated with prolonging cell proliferation and development of cancer.

### 6.3. Cell Cycle

Cell cycle progression is tightly regulated by the interaction between cyclins and cyclin-dependent kinases (CDKs). This interaction results in the selective phosphorylation of target proteins involved in regulatory stages of the cell cycle.

Tax-expressing cells seem to show disruptions in cyclin-CDK complex formation. In general, there appears to be a stronger progression of HTLV-1-infected cells through the G1 phase into the S phase [125]. This can be correlated with the upregulation of cyclins D2 and E and the corresponding increase in D2 binding CDK partners, Cdk4/6 and Cdk2 following Tax expression in these cells [126]. Interestingly, Tax directly binds to cyclin D3, D4, and Cdk4 and apparently increases stability of cyclin D/Cdk4 complex formation [127]. Tax has been reported to activate expression of cdk2 and cdk4 in infected cells [128] and downregulate expression of CDK inhibitors like p18 and p19 [129]. The protein also causes repression of cylin A [130], which is required for cells to exit mitosis after one round of chromosome replication [131] thus promoting enhanced DNA replication.

An important phosphorylation target of the cyclin-CDK complexes is the retinoblastoma protein (Rb) that binds transcription factor E2F in a dephosphorylated state. When phosphorylated by cyclin-CDK complexes, it releases E2F that allows transcription of genes required for the progression from the S to G2 phase [14]. Tax disrupts the formation of Rb-E2F complexes by physically degrading dephosphorylated Rb, promoting its phosphorylation and release of E2F, thus pushing the cell into S phase [132–134]. There is a close association between miRNA expression and alteration in the cell cycle progression. Compelling evidence also points at a markedly altered miRNA expression profile in HTLV-1-infected, ATL, and noninfected cells suggesting a probable association between Tax-induced miRNA expression and rate of cell cycle progression.

### 6.4. DNA Abnormalities

Chromosomal abnormalities associated with Tax-expressing HTLV-1 infection range from deletions, duplications, translocations, and chromosomal rearrangements to aneuploidy [69, 135–140]. Although Tax has not been directly implicated in DNA damage [14, 141], it has been suggested to indirectly promote DNA aberrations through prevention of DNA repair mechanisms that would result in accumulation of mutations [142]. Like most multistage carcinogenic events, virus-induced carcinogenesis is also associated with multiple chromosome defects. A systematic eight-step genetic event sequence has been described by Okamoto et al. (1989) detailing the progression of an HTLV-1-infected cell into a state of cancer [143].

Suppression of antiapoptotic and antitumorigenic factors has been extensively associated with Tax expression in HTLV-1-infected cells. A downregulation of tumor suppressor gene p53 has been observed in 40% of ATL patients [144, 145]. Changes in expression levels of other tumor suppression factors like p15, p16, and Rb cell cycle regulators have also been observed in a number of ATL cases [146–148]. This indicates a strong relationship between suppression of DNA damage repair mechanisms by Tax and transformation of infected cells into leukemia. Some of this suppressive activity could probably be in association with the siRNA pathway that also interacts with chromosomes preventing DNA damage repair.

### 6.5. Histone Modifications

Eukaryotic histone proteins assemble across the entire length of DNA closely associating with it and packaging it into nucleosomal structures within the nucleus. Histone proteins undergo a variety of posttranslational modifications (viz., acetylation, ubiquitylation, methylation, phosphorylation, and SUMOylation) in order to assist in processes such as DNA transcription, replication, and repair.

**Figure 3:**
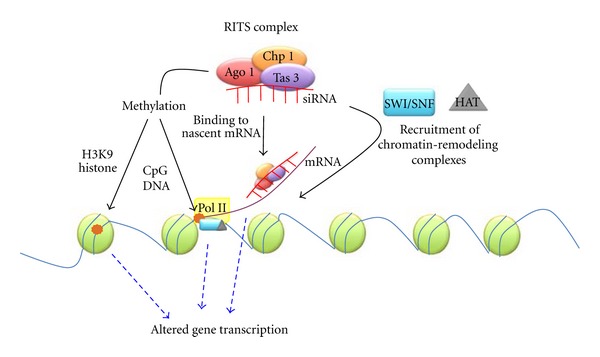
Model for the chromatin remodeling by siRNA/miRNA. siRNA in association with the RNA-induced transcriptional silencing complex can alter the rate of chromosomal transcription in a number of ways. It can directly bind nascent mRNA being transcribed from centromeric heterochromatin preventing its translation. The Ago 1/2 protein of the RITS complex can recruit transcription factors and chromatin-remodeling complexes like SWI/SNF that in turn cause assembly of histone modifying enzymes (viz., HATs, HDACs, etc.). The RITS complex can also recruit methylating enzymes that can either directly methylate CpG regions of centromeric DNA or H3K9 regions on histones associated with the chromosome.

Tax is known to interact with cAMP response-element-binding protein (CREB) at the enhancer region of viral cAMP response element (CRE) that promotes recruitment of coactivators—CBP (CREB binding protein) and p300 [39]. *In vitro* studies indicate that these coactivators have histone acetylase activity that aids in HTLV-1 transcription [149]. CHIP analysis of the integrated HTLV-1 proviral site in infected SLB1 T cells revealed the recruitment of a number of factors namely, CREB, CREB-2, ATF-1, ATF-2, c-Fos, c-jun, p300, P/CAF, CBP, and Tax at the promoter region. The authors also observed histone H3 and H4 acetylation within the proviral genome of these cells. Interestingly, histone deacetylases (HDACs) were also found to localize at the promoter region and inhibition of the HDACs with specific antagonists-enhanced acetylation states of H4 and an increase in HTLV-1 RNA transcription (see Figure 3).

Lu et al. (2004) showed that the HDAC-1 directly associated with the inactive Tax and not with its transactivated form. Biotinylated chromatin pull down assays revealed that Tax promoted dissociation of HDAC-1 with the promoter region of HTLV-1 [150] suggesting modulation of HDAC recruitment by tax as a means of transcriptional regulation.

Tax promotes its own transactivation by associating with BRIG1 components of the ATP-dependent SWI/SNF chromatin remodeling complex downstream of Pol II [151]. Tax, therefore, appears to modulate the transcriptional state of chromatin by strongly associating with histone modifying proteins that effect alterations between euchromatin and heterochromatin during an HTLV-1 infection both *in vivo* and *in vitro*.

In addition to the oncoprotein, other HTLV-1 associated proteins can also be associated with the miRNA pathway and contribute to chromatin remodeling. Animal studies with mutated forms of accessory genes like p12, p30, Rex, p13, and HTLV-1 basic leucine zipper factor (HBZ) using HTLV-1 infectious clones revealed that these proteins are required to establish *in vivo* latency [152–155]. Rex protein appears to interact with Dicer preventing it from cleaving siRNAfrom shRNA and thus acting as a suppressor of RNA silencing [37]. Although there is a certain degree of sequence similarity between Rex and Tax proteins, it is not very clear if this confers a similar interaction property with host cellular proteins, like those involved in miRNA processing [156, 157]. The p30 accessory protein can physically bind tax and Rex mRNAs and retain both transcripts in the nucleus, preventing their translation in the cytoplasm. This in turn may influence the rate at which viral transcription occurs and could cause a switch from active to dormant states of viral replication [158]. In addition, p30 also interacts with CREB-binding protein (CBP) and p300, altering rate of transcription of the long terminal repeat region of HTLV-1 as well as CBP and p300-dependent cellular genes [159].

## 7. Concluding Notes

miRNA-mediated transcriptional gene silencing is rapidly gaining significance as one of the major modes of chromatin remodeling and alteration of the rate of gene expression in cells. From past and current literature, it is evident that viruses and their proteins actively and effectively utilize the miRNA machinery to alter cellular and viral gene expression, ultimately influencing the progression of disease. Chronic viruses that integrate their genome into that of the hosts' exploit small RNA pathways for establishing latency or increasing the rate of viral protein expression to ultimately transform cells and maximize survival.

**Figure 4:**
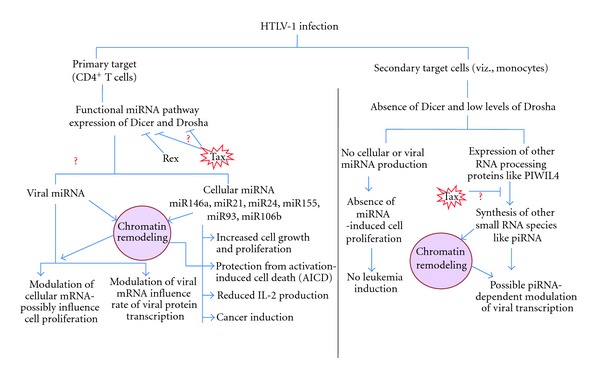
Differential miRNA expression and its impact on chromatin remodeling in primary and secondary target cells during HTLV-1 infection.

Although protein families involved in siRNA-mediated epigenetic silencing pathways are relatively conserved between species and cell types, there exists a noticeable alteration in the level of these proteins. Also, the presence of alternate stages in pathways like RdRP-dependent siRNA amplification in *Saccharomyces cerevisiae *and alternate small RNA species like PIWI-associated RNA being predominantly active in some cell types like germline cells needs to be taken into account. As discussed in the paper, a huge variation exists in miRNA expression profiles of not only different stages of viral infection but also different cell types. The differential progression of viral infection in primary and nonprimary target cells has always been an intriguing aspect of HTLV-1 infection studies. The refractile nature of some cell types in contrast to the ability of others to support viral transcription has been attributed among other factors to the difference in miRNA processing proteins that make certain cell types less supportive of miRNA expression and hence of chromatin manipulation through the siRNA/miRNA-mediated pathways.  Figure 4 represents a schematic outline of inherent differences in miRNA expression in primary and secondary target cells of HTLV-1 that could contribute to differing outcomes in both. Deciphering factors that dictate this cell type preference and specificity can go a long way in the advancement of HTLV-1 research and pave the way for developing novel and more effective therapeutic strategies.
